# Enhancing meloxicam efficacy in acute and chronic inflammatory pain: a novel dose-sparing approach using ultramicronized palmitoylethanolamide

**DOI:** 10.1007/s10787-026-02380-6

**Published:** 2026-10-06

**Authors:** Samuele Trisolini, Alessandra Toti, Elena Lucarini, Lorenzo Di Cesare Mannelli, Carla Ghelardini, Laura Micheli

**Affiliations:** Department of Neuroscience, Psychology, Drug Research and Child Health – NEUROFARBA - Pharmacology and Toxicology Section, University of Florence, Viale Pieraccini 6, 50139 Florence, Italy

**Keywords:** Inflammatory pain, Ultramicronized palmitoylethanolamide, Meloxicam, Sparing effect, PPAR-α, LPS

## Abstract

Nonsteroidal anti-inflammatory drugs (NSAIDs), such as meloxicam, are widely used for the management of inflammatory pain in human and veterinary medicine, although their clinical utility is limited by dose-dependent adverse effects. Strategies aimed at reducing NSAID dosage while preserving analgesic efficacy are therefore warranted. We investigated whether ultramicronized palmitoylethanolamide (PEAum), an endogenous prohomeostatic lipid amide with improved bioavailability, could enhance the antinociceptive effects of meloxicam in preclinical models of acute and chronic inflammatory pain. Acute pain was induced by intraplantar lipopolysaccharide (LPS) injection, while chronic pain was induced by intra-articular injection of Complete Freund’s Adjuvant (CFA). Mechanical hyperalgesia and allodynia were measured by the Paw pressure and von Frey tests, respectively. In the LPS model, PEAum confirmed its superior anti-hyperalgesic profile compared with micronized and native PEA. PEAum (10 mg/kg) markedly enhanced the analgesic activity of low-dose meloxicam (3 mg/kg), with the combination fully reversing inflammatory hypersensitivity and outperforming high-dose meloxicam (30 mg/kg). In the CFA model, the same association completely counteracted mechanical hyperalgesia, reproducing the antalgic effect of high-dose meloxicam. A pharmacodynamic study demonstrated that pharmacological blockade of PPAR-α with the selective receptor antagonist GW6471 (2 mg/kg) significantly reduced, but did not abolish, the analgesic effect of the combination, suggesting both PPAR-α-dependent and independent mechanisms. Overall, the combined treatment produced a greater-than-additive antinociceptive effect, demonstrating a synergistic interaction between PEAum and meloxicam in both acute and chronic inflammatory pain. This synergy enables substantial meloxicam dose-reduction while maintaining full analgesic activity, supporting a promising dose-sparing strategy for inflammatory pain management.

## Introduction

Nonsteroidal anti-inflammatory drugs (NSAIDs) are among the most widely prescribed pharmacological agents for the management of inflammatory pain in both human and veterinary medicine. Their therapeutic efficacy relies on the inhibition of cyclooxygenase (COX) enzymes and the resulting suppression of prostaglandin synthesis, key mediators of peripheral sensitization and inflammatory hyperalgesia (Vane and Botting, 1998). Despite their effectiveness, NSAIDs are intrinsically limited by dose-dependent adverse effects, including gastrointestinal mucosal damage, renal dysfunction, and increased cardiovascular risk, particularly during chronic administration (Bjarnason et al. 2018) and in frail subjects, such as older patients or those with pre‑existing comorbidities. Even COX-2 preferential agents such as meloxicam, although associated with improved tolerability, do not eliminate these risks (For the International NSAID Consensus Group et al. 2015; Cooper et al. 2019).

Meloxicam is extensively used in veterinary medicine for the treatment of acute and chronic inflammatory pain (Gruen et al. 2019; Steagall et al. 2026) and is also employed in selected human indications, including rheumatic and degenerative joint disorders, as well as postoperative pain (Bekker et al. 2018). However, as with other NSAIDs, achieving robust analgesia often requires doses that may increase the probability of systemic adverse events. Therefore, a major translational challenge in inflammatory pain management is to enhance NSAID analgesic efficacy while reducing the required dosage. A pharmacological strategy capable of potentiating NSAID-induced antinociception could allow dose reduction without compromising therapeutic outcomes, thereby improving the overall safety profile.

One possible approach is the combination of NSAIDs with endogenous lipid mediators that modulate inflammation and nociceptive processing through complementary mechanisms. Palmitoylethanolamide (PEA) is an endogenous fatty acid amide belonging to the N-acylethanolamine family, physiologically produced “on demand” in response to tissue injury and inflammation. First described for its anti-inflammatory properties in the mid-twentieth century, PEA is now recognized as a pleiotropic modulator of neuroinflammation and pain signalling (Skaper et al. 2014).

PEA is not a classical analgesic but a pro-homeostatic lipid mediator that modulates inflammatory and nociceptive processes through multiple pathways. Its actions derive from both direct receptor engagement and indirect amplification of endogenous lipid-signaling pathways, enabling coordinated control of neuroimmune activation. The principal direct target is peroxisome proliferator-activated receptor-α (PPAR-α), a nuclear receptor governing transcriptional programs involved in inflammatory responses and lipid metabolism (Lo Verme et al. 2005). Through PPAR-α activation, PEA downregulates NF-κB–driven pro-inflammatory mediators, reduces mast cell degranulation, and attenuates astrocytic and microglial activation, key drivers of peripheral and central sensitization (Lo Verme et al. 2005; Skaper et al. 2014). In parallel, PEA exerts indirect receptor-level effects, most notably by enhancing the activity of endogenous cannabinoids and thereby potentiating signaling at CB1 and CB2 receptors through an entourage mechanism. PEA also modulates TRPV1 channel responsiveness and influences additional lipid-sensitive GPCRs, further contributing to the resolution of neuroinflammation (Petrosino and Di Marzo 2017).

Its endogenous nature, multimodal mechanism, and lack of direct interference with prostaglandin synthesis confer an excellent safety profile and make PEA an attractive candidate for combination therapies aimed at enhancing analgesic efficacy while reducing NSAID-associated toxicity.

However, the clinical and experimental efficacy of PEA is strongly influenced by its bioavailability. Native PEA shows limited oral absorption, which can constrain its pharmacodynamic potential. To address this limitation, micronization techniques were developed to increase particle surface area, thereby enhancing dissolution kinetics and facilitating tissue distribution. Among these formulations, ultramicronized PEA (PEAum) provides faster and more consistent pharmacological responses, likely due to superior absorption and systemic bioavailability following oral administration (Petrosino et al. 2018). Enhanced bioavailability translates into a more rapid onset and greater magnitude of analgesic activity, making the ultramicronized formulation particularly suitable for combination approaches (Impellizzeri et al. 2014).

From a mechanistic standpoint, combining meloxicam with PEAum could represent a multimodal analgesic strategy targeting distinct yet complementary components of inflammatory pain. Meloxicam primarily modulates prostaglandin-dependent pathways (Davies and Skjodt 1999), whereas PEA regulates neuroimmune activation through PPAR-α and indirect modulation of CB1/CB2 and TRPV1 signaling (Rankin and Fowler 2020). This convergence on separate but interacting nociceptive circuits raises the possibility of synergistic interactions, in which the combined effect might exceed the sum of the individual components. If confirmed, such synergy would support a dose-sparing strategy, allowing effective restoration of nociceptive thresholds with substantially lower meloxicam doses.

In this context, the present study was designed to investigate whether PEAum enhances the antalgic efficacy of meloxicam in two well-established models of inflammatory pain, that capture distinct temporal dimensions of the nociceptive process: lipopolysaccharide (LPS)-induced intraplantar inflammatory hypersensitivity (representing an acute inflammatory pain state) and Complete Freund's Adjuvant (CFA)-induced articular inflammation (modelling chronic inflammatory pain). We hypothesized that combining PEAum with low-dose meloxicam could yield synergistic antinociceptive effects, thereby enabling substantial meloxicam dose-reduction without compromising its analgesic efficacy.

## Materials and methods

### Animals

Male Sprague Dawley rats (Charles River, Calco, Italy) weighing approximately 220−250 g at the beginning of the experimental procedure were used. Animals were housed in Ce.S.A.L (Centro Stabulazione Animali da Laboratorio, University of Florence, Italy) and used at least 1 week after their arrival. Four rats were housed per cage; animals were fed a standard laboratory diet and tap water ad libitum and kept at 23 ± 1 °C with a 12 h light/dark cycle, light at 7 a.m.

### LPS-induced paw inflammatory pain

Inflammatory pain was induced by intraplantar injection of lipopolysaccharide (LPS; 10 μg in 50 μl of 0.9% NaCl; Sigma-Aldrich) into the plantar surface of the rat hind paw under isoflurane anaesthesia, as previously described (Inceoglu et al. 2006). Control animals received an equivalent volume of vehicle (0.9% NaCl). Ten minutes prior to LPS injection, animals were orally administered ultramicronized palmitoylethanolamide (hereafter referred to as PEAum), micronized palmitoylethanolamide (hereafter referred to as PEAm), native palmitoylethanolamide (hereafter referred to as PEAnat), or meloxicam or vehicle alone.

### CFA-induced articular pain

Articular damage was induced by an injection of Complete Freund’s Adjuvant (CFA) (Merck, Milan, Italy) into the tibio–tarsal joint (Butler et al. 1992; Micheli et al. 2019). Briefly, rats were lightly anesthetized with 2% isoflurane. After sterilizing the skin of the left leg with 75% ethyl alcohol, the lateral malleolus was located by palpation, and a 28-gauge needle was inserted vertically to penetrate the skin and turned distally for insertion into the articular cavity at the gap between the tibiofibular and tarsal bone until a distinct loss of resistance was felt. A volume of 50 μL of CFA was then injected (left paw, ipsilateral). Control rats received 50 μL of saline solution in the tibio–tarsal joint. Behavioral tests were performed when articular pain was well established (starting 7 after CFA injection).

### Treatments

In the LPS-induced inflammatory pain model, meloxicam, PEAum, PEAm, and native PEA were administered orally, either alone or in combination, after suspension in 1% sodium carboxymethylcellulose (CMC).

For the pharmacodynamic study, the selective PPAR-α antagonist GW6471 (2 mg/kg, i.p.) was used (Lucarini et al. 2022). GW6471 was dissolved in 10% DMSO/5% Tween 20/saline and administered 15 min before drug treatment and 25 min prior to LPS injection.

In the CFA-induced rheumatoid arthritis model, meloxicam and PEAum were suspended in 1% CMC and administered orally, either alone or in combination, on day 7 after CFA injection.

### Paw pressure test

The nociceptive threshold was determined with an analgesimeter (Ugo Basile, Varese, Italy). Briefly, constantly increasing pressure was applied to a small area of the dorsal surface of the hind paw using a blunt conical mechanical probe. Mechanical pressure was increased until vocalization or withdrawal reflex occurred, while the rats were lightly restrained. Vocalization or withdrawal reflex thresholds are expressed in grams (g). In this study, an arbitrary cut-off value of 100 g was used. Data were collected by an observer who was blinded to the treatment protocol (Micheli et al. 2021).

### Von Frey test

The mechanical allodynia was measured with an electronic von Frey hair unit (Ugo Basile, Varese, Italy). Briefly, the rats were in 20 × 20 cm Plexiglas boxes furnished with a metallic mesh floor 20 cm above the bench. After 5 min of adaptation, a force ranging from 0 to 50 g (accuracy of 0.2 g) was applied to estimate the withdrawal threshold. A punctuate stimulus was applied to the mid-plantar area of each anterior paw from below the mesh floor through a plastic tip and the withdrawal threshold was automatically displayed on the screen. The paw sensitivity threshold was defined as the lowest pressure necessary to provoke a vigorous and instantaneous withdrawal reflex of the paw. The measure was repeated 3 times with an interval of 5 s and then expressed as a mean (Micheli et al. 2019).

### Statistical analysis

The researchers were blind to all treatments. Data are expressed as mean ± standard error of the mean (SEM) of six [or eight] rats per group. The area under the curve (AUC) from 0 to 60 min was calculated using the trapezoidal rule, with time expressed in hours; thus, a 15-min interval corresponded to 0.25 h. The percentage of pain inhibition was calculated as follows: 100 × (treatment − vehicle)/(control − vehicle).

Data were analyzed using a generalized linear model (GLM) followed by Tukey–Kramer-adjusted post hoc comparisons.

For the synergy analysis, a factorial two-way ANOVA was performed according to the approach described by Slinker (Slinker 1998) to determine whether the effect of the combined administration of the study compounds exceeded the sum of the individual effects of each treatment. The analysis was conducted on raw data or on log-transformed data, depending on whether mean responses or percentage of pain inhibition were evaluated. All statistical analyses were conducted using SAS software (version 9.4; SAS Institute, Cary, NC, USA). A *p* value < 0.05 was considered statistically significant.

## Results

### Pain-relieving effect of different PEA formulations against acute inflammatory pain

**Fig. 1:**
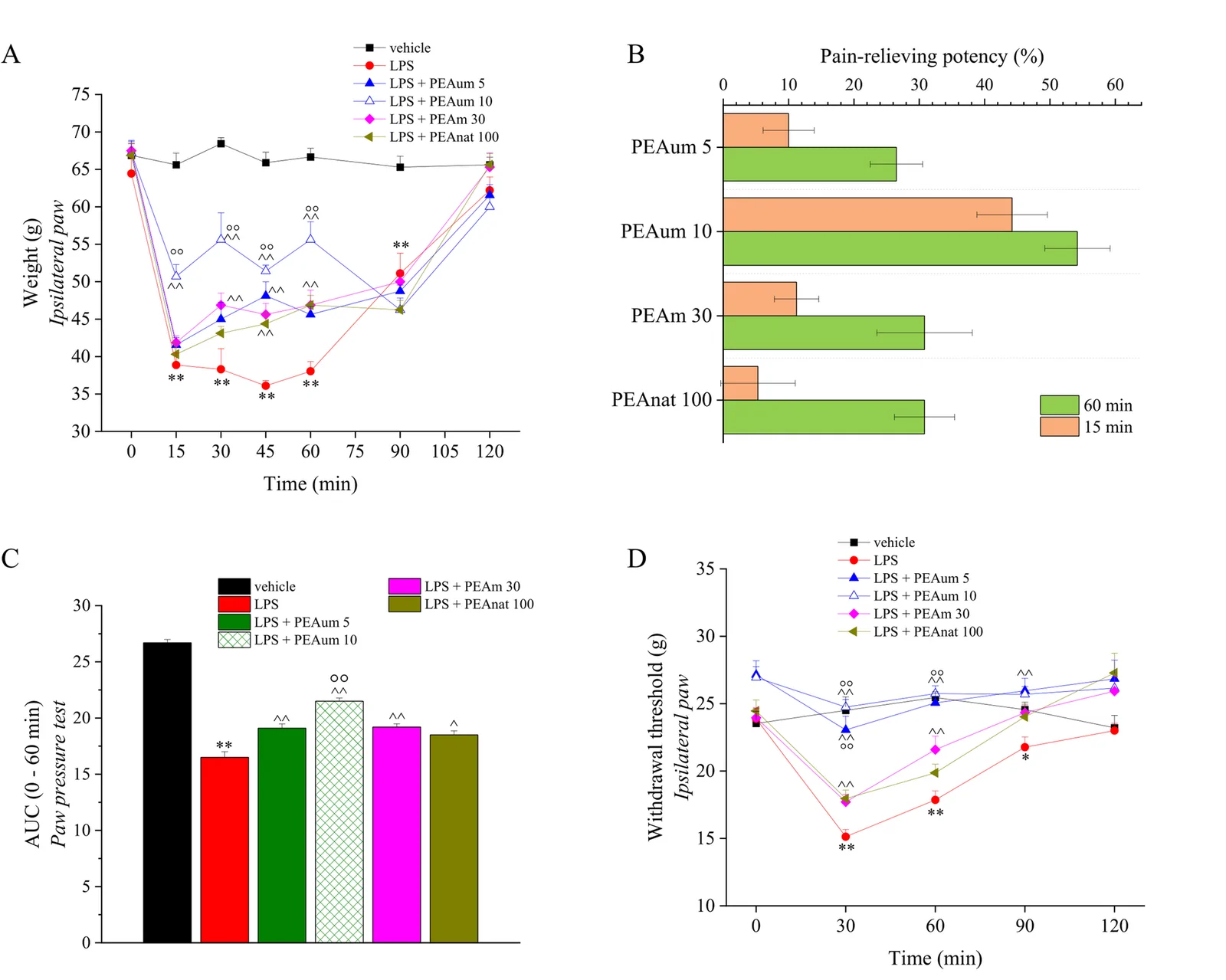
Antinociceptive effects of different PEA formulations in LPS-induced acute inflammatory pain model. Lipopolysaccharide (LPS; 10 μg in 50 μL of 0.9% NaCl) was intraplantarly injected to induce acute inflammatory pain. Nociceptive thresholds were evaluated before (0 min) and at different time points after LPS injection (15–120 min), covering the development and resolution of the pain condition. Ten minutes prior to LPS injection, different formulations of palmitoylethanolamide (PEA) were suspended in 1% CMC and orally administered: ultramicronized PEA (PEAum, 5 and 10 mg/kg), micronized PEA (PEAm, 30 mg/kg), and native PEA (PEAnat, 100 mg/kg). Time-course analyses show the pain-relieving effects of the different PEA formulations on mechanical hyperalgesia and allodynia, assessed by Paw pressure and von Frey tests (**A** and **D**, respectively). The overall antinociceptive efficacy of the treatments was further evaluated by determining the percentage of pain inhibition at 15 min and 60 min (**B**) and by calculating the area under the curve (AUC) over the first 60 min after administration (**C**). The effects of the different PEA formulations on allodynia were assessed by von Frey test (**D**). Data are expressed as mean ± S.E.M of 8 rats per group. Statistical analysis was performed by GLM followed by Tukey-Kramer post hoc test for multiple comparisons. *p < 0.05 and **p < 0.0001 vs vehicle; ^*p* < 0.05 and ^^*p* < 0.0001 vs LPS; °°*p* < 0.05 vs PEAm/PEAnat

Intraplantar LPS injection induced a robust inflammatory pain state, as evidenced by a marked reduction in nociceptive thresholds compared with control animals, starting at 15 min, persisting up to 60 min, and then gradually declining. This effect was consistently observed across both mechanical sensitivity paradigms—hyperalgesia (Paw pressure test; Fig. 1A) and allodynia (von Frey test; Fig. 1D)—supporting the establishment of a stable inflammatory pain condition (*p* < 0.0001 versus controls at all time points).

The anti-hyperalgesic efficacy of different PEA formulations was first evaluated against a noxious mechanical stimulus. Ultramicronized, micronized, and native PEA (PEAum, PEAm, and PEAnat, respectively) were tested. In the Paw pressure test (Fig. 1A), all formulations significantly attenuated LPS-induced hyperalgesia compared with vehicle-treated animals, although with different degrees of efficacy. Among these, PEAum showed the highest pain-relieving profile, increasing the weight bearing on the LPS-injected paw in a dose-dependent manner. Notably, PEAum at 10 mg/kg outperformed PEAm (30 mg/kg) and PEAnat (100 mg/kg) and matched their effects at just 5 mg/kg. Moreover, PEAum (10 mg/kg) was the only formulation showing significant activity as early as 15 min (*p* < 0.0001 versus vehicle). When expressed as percentage pain inhibition at the first time point (15 min) and before the physiological decline of the model (60 min), PEAum (10 mg/kg) was confirmed as the most effective formulation (Fig. 1B), achieving >50% inhibition of LPS-induced pain.

To provide an integrated measure of the antinociceptive response over time, the area under the curve (AUC) was calculated over the first hour following treatment (Fig. 1C). Consistent with the time-course analysis, PEAum (10 mg/kg) showed the greatest overall efficacy among the PEA formulations.

A similar pattern was observed in a measure of mechanical allodynia performed with the von Frey test at multiple time points (Fig. 1D), where PEAum exerted a more pronounced anti-allodynic effect compared with the other formulations, confirming its superior efficacy across different modalities of mechanical sensitivity.

### Pain-relieving effect and synergistic interaction of PEAum and meloxicam against acute inflammatory pain

Given its superior antinociceptive efficacy among the tested formulations described before, PEAum was co-administered at two doses (5 and 10 mg/kg) with a low-dose meloxicam (3 mg/kg) to evaluate its potential adjunctive effect in the same LPS-induced acute inflammatory pain model. The effects of the associations were also compared with a high-dose meloxicam (30 mg/kg).

**Fig. 2:**
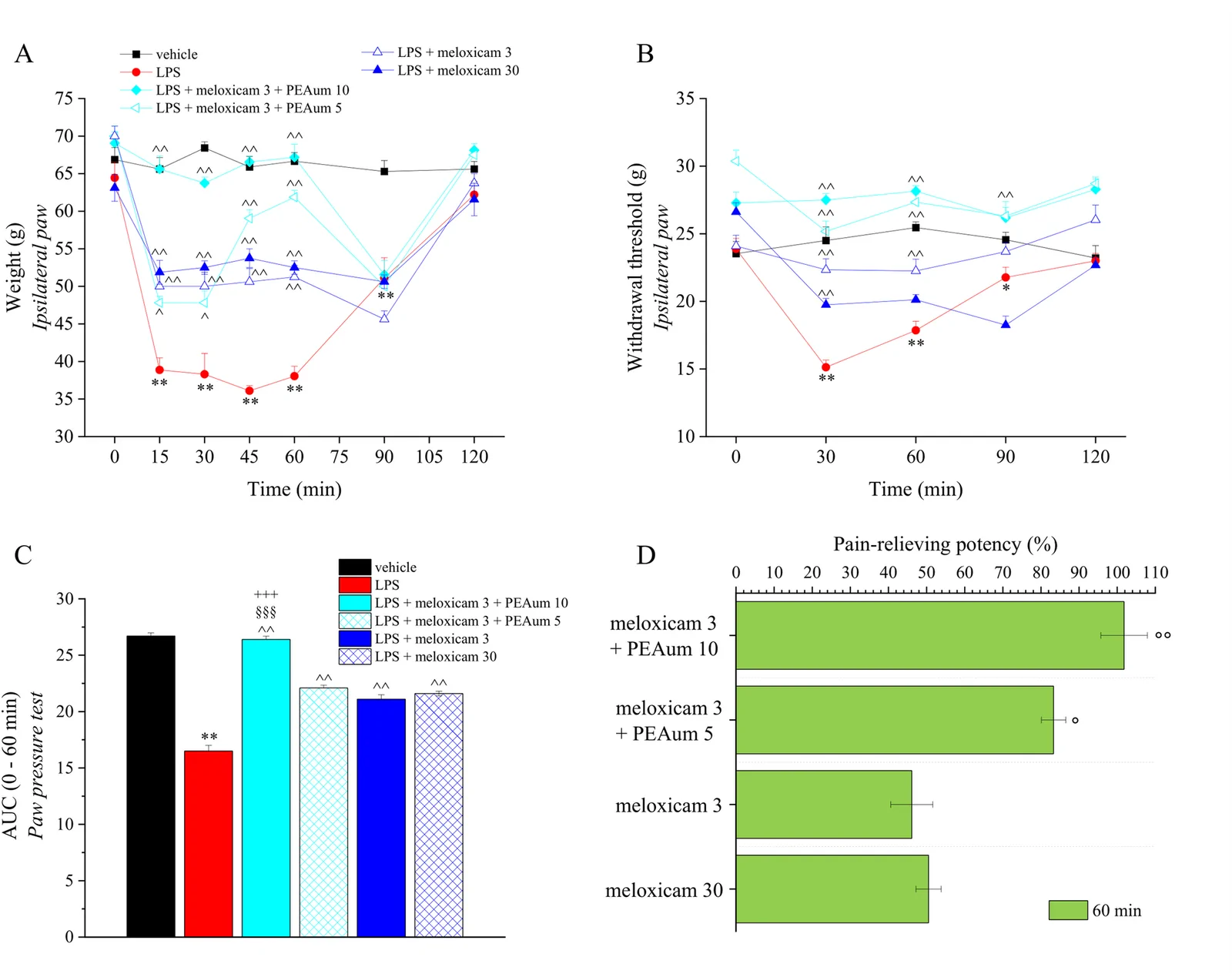
Pain-relieving effect of PEAum–meloxicam combination in LPS-induced acute inflammatory pain. Lipopolysaccharide (LPS; 10 μg in 50 μL of 0.9% NaCl) was intraplantarly injected to induce acute inflammatory pain. Nociceptive thresholds were evaluated before (0 min) and at different time points after LPS injection (15–60 min). Ten minutes prior to LPS injection, ultramicronized palmitoylethanolamide (PEAum, 5 and 10 mg/kg), in combination with meloxicam (3 mg/kg), was suspended in 1% CMC and orally administered. Meloxicam 3 and 30 mg/kg was also tested alone. Mechanical hyperalgesia and allodynia were assessed by the Paw pressure test (**A**) and the von Frey test (**B**), respectively. Time-course analyses show the effects of the combination treatments and meloxicam. The overall antinociceptive efficacy was further evaluated by calculating the area under the curve (AUC) over the first 60 min after administration (**C**) and by determining the percentage of pain inhibition at 60 min measured by paw pressure test (**D**). Data are expressed as mean ± S.E.M of 8 rats per group. Statistical analysis was performed by GLM followed by Tukey-Kramer post hoc test for multiple comparisons. *p < 0.05 and **p < 0.0001 vs vehicle; ^*p* < 0.01 and ^^*p* < 0.0001 vs LPS; ^§§§^*p* < 0.0001 vs meloxicam both 3 and 30; +++*p* < 0.0001 vs meloxicam 3 + PEAum 5; °°*p* = 0.0001 and °°°*p* < 0.0001 vs meloxicam 30

In the paw pressure test (Fig. 2A), both combination treatments markedly counteracted the LPS-induced reduction in nociceptive threshold, indicating a strong attenuation of mechanical hyperalgesia. The higher dose of PEAum in combination with meloxicam produced the most pronounced effect, significantly surpassing the effect of meloxicam 3 mg/kg (*p* < 0.0001 at all-time points) and completely reversing hyperalgesia and restoring nociceptive thresholds to levels comparable to those of control animals at multiple time points up to 1 h (*p* = 1 versus LPS at all time points except 30 min). The lower dose of PEAum in combination produced a less marked effect; however, nociceptive thresholds remained higher than those observed with meloxicam alone, particularly at 45 min (*p* = 0.0008) and 60 min (*p* = 0.0003). Meloxicam, administered alone, both at the doses of 3 mg/kg and 30 mg/kg was partially effective.

The findings were confirmed in the von Frey test (Fig. 2B), where both combinations effectively counteracted LPS-induced mechanical allodynia, although without a clear dose–response relationship.

Further analysis on AUC over the first hour of Paw pressure test (Fig. 2C) showed that the combination of PEAum (10 mg/kg) with meloxicam achieved the greatest overall efficacy, being significantly more effective than meloxicam used singly at both 3 and 30 mg/kg (*p* < 0.0001 for both comparisons). In contrast, the combination with PEA um lower dose displayed an intermediate effect, comparable to meloxicam alone. Notably, the combination PEAum (10 mg/kg) + meloxicam (3 mg/kg) achieved an AUC value indistinguishable from that of the control (vehicle-injected) group (*p* = 1 versus vehicle), indicating full normalization of LPS-induced hypersensitivity.

Consistently, the percentage of pain inhibition at 60 min (Fig. 2D) highlighted the superior efficacy of the combination treatments, irrespective of the PEAum dose, even compared with high-dose meloxicam alone.

Importantly, synergy analysis based on this endpoint showed that PEAum (5 and 10 mg/kg) enhanced the effect of low-dose meloxicam in a synergistic manner, with the observed response exceeding the expected sum of the individual effects (*p* < 0.0001 and *p* = 0.0014, respectively).

### Involvement of PPAR-α in the antinociceptive effect of the PEAum–meloxicam combination

The contribution of PPAR-α signaling to the antinociceptive effect of the PEAum–meloxicam combination was assessed by pharmacological blockade with GW6471 (2 mg/kg), a selective PPAR-α antagonist.

**Fig. 3:**
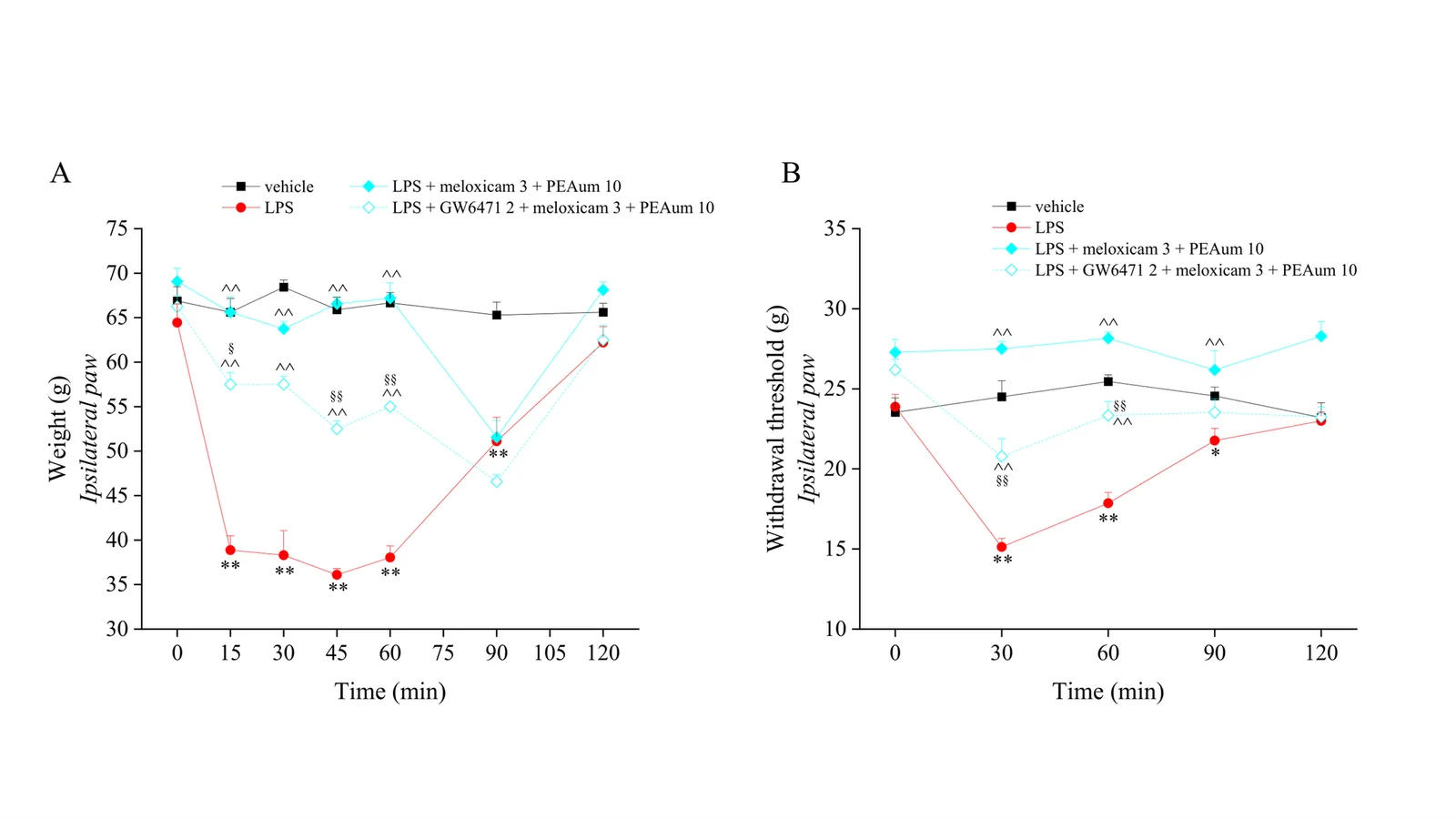
Effect of PPAR-α blockade on the antinociceptive activity of the PEAum–meloxicam combination. Lipopolysaccharide (LPS; 10 μg in 50 μL of 0.9% NaCl) was intraplantarly injected to induce acute inflammatory pain. Nociceptive thresholds were evaluated before (0 min) and at different time points after LPS injection (15–60 min). The PPAR-α antagonist GW6471 (2 mg/kg) was dissolved in 10% DMSO/5% Tween 20/saline and administered 15 min before PEAum 10 mg/kg + meloxicam 3 mg/kg treatment and 25 min prior to LPS injection. Mechanical hyperalgesia and allodynia were assessed by paw pressure test (**A**) and von Frey test (**B**), respectively. Data are expressed as mean ± S.E.M of 8 rats per group. Statistical analysis was performed by GLM followed by Tukey-Kramer post hoc test for multiple comparisons. *p < 0.05 and **p < 0.0001 vs vehicle; ^^*p* < 0.0001 vs LPS; ^§^*p* < 0.05 and ^§§^*p* < 0.0001 vs meloxicam 3 + PEAum 10

In the paw pressure test (Fig. 3A), the combination of PEAum and meloxicam markedly increased the animal’s pain thresholds, an effect significantly attenuated by GW6471 pretreatment.

Comparable findings were obtained in the von Frey test (Fig. 3B). While the combination strongly reduced mechanical allodynia, the effect was significantly blunted by GW6471 pre-treatment. Residual activity remained compared with LPS-treated animals, but withdrawal thresholds were consistently lower than those observed with the combination alone. Overall, PPAR-α blockade significantly weakened the antinociceptive efficacy of the PEAum + meloxicam combination across both paradigms (hyperalgesia and allodynia), supporting the involvement of PPAR-α-dependent mechanisms in the effect of the combination.

### PEAum synergistically potentiates antinociceptive effects of meloxicam in chronic inflammatory pain

To extend these findings from an LPS-induced acute inflammatory pain model to a chronic setting, the effects of meloxicam with or without PEAum were evaluated in CFA-induced joint pain. Behavioural assessment was performed 7 days after CFA injection, when a stable and sustained reduction in nociceptive threshold compared with the control group confirmed the establishment of a chronic pain state.

**Fig. 4:**
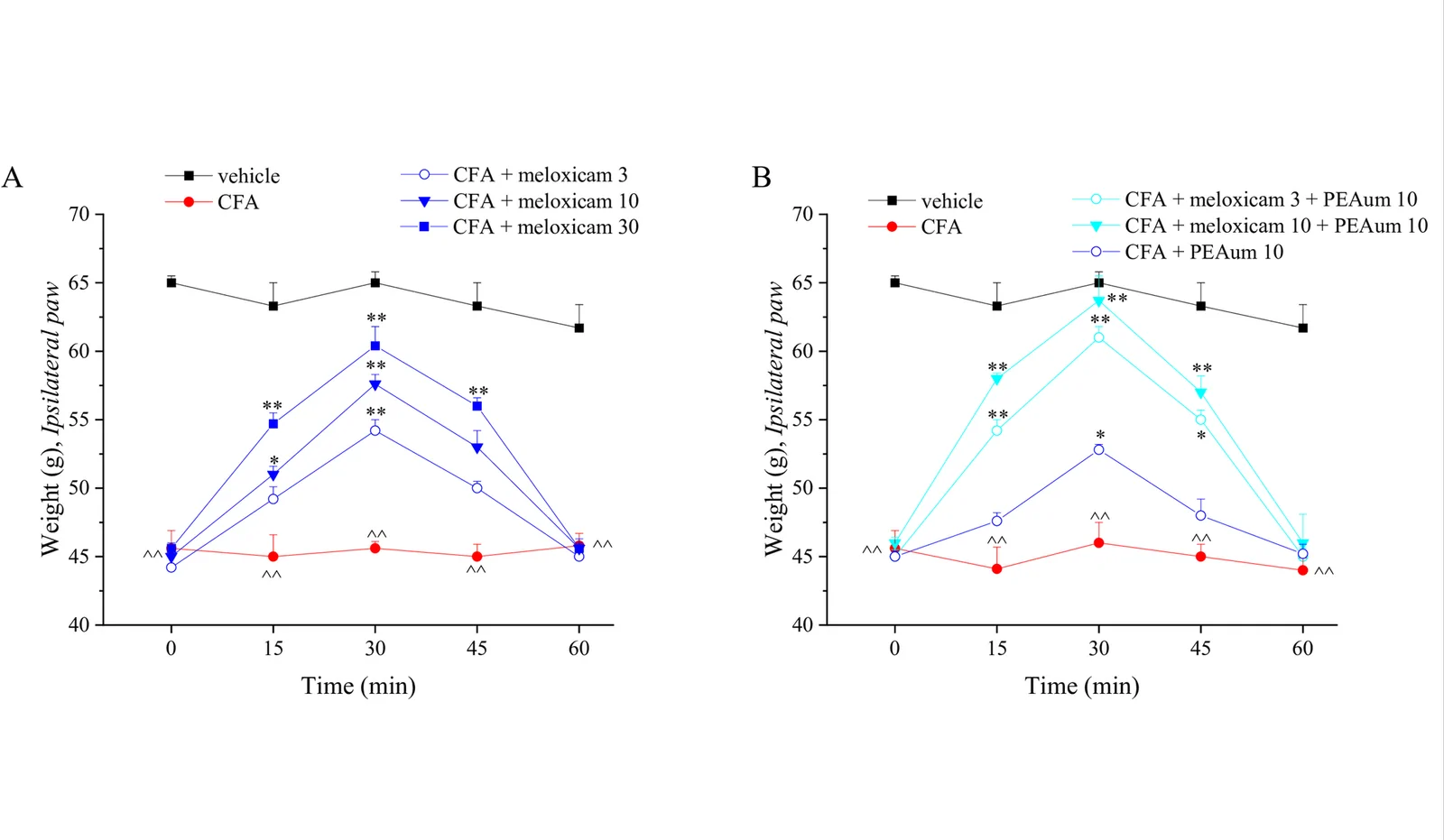
Antinociceptive effects of meloxicam with or without PEAum in a model of CFA-induced chronic inflammatory pain. CFA (50 μL; Sigma Aldrich) was intra-articularly injected to induce chronic inflammatory joint pain. Meloxicam (3, 10, and 30 mg/kg) and ultramicronized palmitoylethanolamide (PEAum, 10 mg/kg), alone or in combination, were suspended in 1% CMC and orally administered when hypersensitivity was well established (day 7). Mechanical hyperalgesia was assessed by the paw pressure test. Time-course analyses show the dose-dependent antinociceptive effect of meloxicam (**A**) and the effects of PEAum alone or in combination with meloxicam (**B**). Data are expressed as mean ± S.E.M of 6 rats per group. Statistical analysis was performed by GLM followed by Tukey–Kramer post hoc test for multiple comparisons. ^^*p* < 0.0001 vs vehicle; *p < 0.01 **p < 0.0001 vs CFA

In the paw pressure test (Fig. 4A), meloxicam (3, 10 and 30 mg/kg) produced a clear dose-dependent antinociceptive effect, peaking at 30 min and declining by 60 min.

The combination with PEAum (10 mg/kg) markedly increased nociceptive thresholds compared with either compound alone (Fig. 4B). Notably, the combination with low-dose meloxicam (3 mg/kg) produced an effect comparable to that of higher meloxicam doses used singly, indicating a clear tenfold potentiation. PEAum alone exerted a detectable but less pronounced effect.

**Fig. 5:**
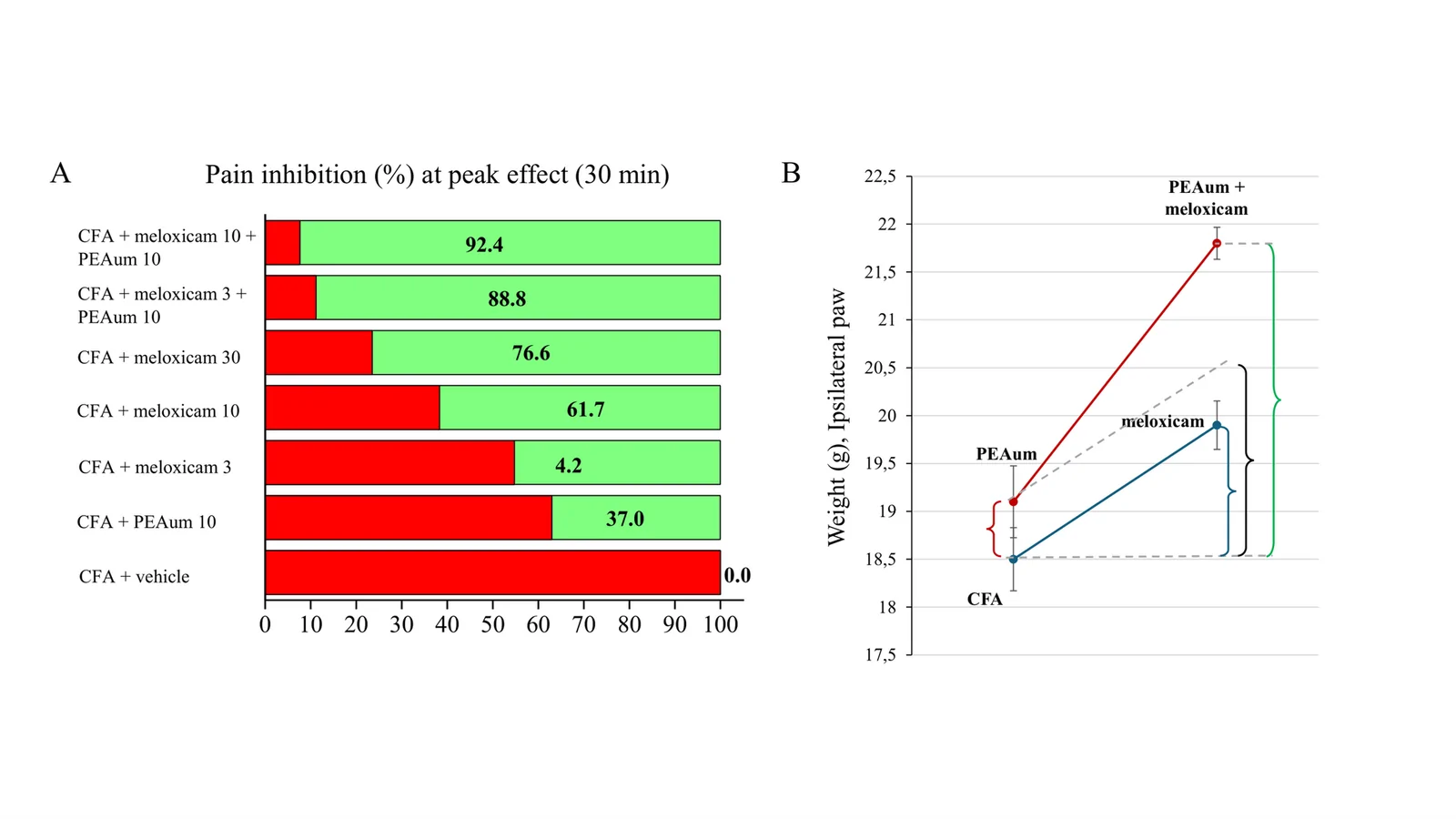
Synergistic interaction between PEAum and meloxicam in CFA-induced chronic inflammatory pain. CFA (50 μL; Sigma Aldrich) was intra-articularly injected to induce chronic inflammatory joint pain. Ultramicronized palmitoylethanolamide (PEAum, 10 mg/kg) and meloxicam (3, 10 or 30 mg/kg) were administered alone or in combination, suspended in 1% CMC. (**A**) Percentage inhibition of inflammatory pain at 30 min (peak effect), assessed by the paw pressure test. The green bars indicate the antinociceptive effect (i.e., percentage of pain inhibition) for each treatment. (**B**) Drug interaction analysis based on AUC mean values, represented according to Slinker 1998. The blue and red brackets represent the effect of meloxicam and PEAum, respectively. In a two-way ANOVA (2×2 factorial analysis), the observed effect (green bracket) was significantly greater than the expected additive effect (black bracket), indicating a synergistic interaction between PEAum and meloxicam

At peak effect (30 min; Fig. 5A), combination treatments produced greater pain inhibition than individual treatments, with PEAum (10 mg/kg) plus meloxicam (3 or 10 mg/kg) achieving the highest levels (92% and 89% pain inhibition, respectively).

Analysis based on AUC values (Fig. 5B) revealed a clear synergistic interaction, with the antinociceptive response to the combination significantly exceeding the expected additive effect of the individual treatments (*p* = 0.045; Fig. 5B).

## Discussion

The present study demonstrates that ultramicronized palmitoylethanolamide (PEAum) markedly enhances the antinociceptive efficacy of meloxicam in both acute and chronic rat models of inflammatory pain. The most relevant finding is that combining PEAum with a low dose of meloxicam produces a synergic analgesic effect, compared to either compound administered alone. This interaction allows for a substantial reduction in NSAID dosage while maintaining full antinociceptive efficacy. Importantly, the effect was consistently observed across two distinct experimental models of acute and chronic pain, i.e., LPS-induced inflammatory pain and CFA-induced joint pain, thereby supporting the robustness and potential translational relevance of the interaction.

NSAIDs such as meloxicam are widely used in both human and veterinary practice due to their well-established efficacy in controlling inflammatory pain. However, their use is significantly limited by dose-dependent adverse effects, including gastrointestinal, renal, and cardiovascular toxicity (Wolfe and Singh 1999; Bjarnason et al. 2018; Arfeen et al. 2024). Among these, gastrointestinal (GI) toxicity represents one of the most clinically relevant limitations. Both short- and long-term NSAID administration can induce damage throughout the GI tract in humans and animals, ranging from asymptomatic mucosal lesions to severe complications such as ulcers, bleeding, and perforation, particularly in predisposed and frail individuals (Bjarnason et al. 2018; Hillier et al. 2025). Notably, the risk of these adverse events is dose-dependent and increases with higher doses and prolonged exposure (Laine 2001). Endoscopic studies indicate that a substantial proportion of NSAID users develop GI lesions, with clinically relevant complications occurring in a non-negligible percentage of chronic users. While the incidence of upper GI complications has declined over time due to improved preventive strategies, growing evidence indicates that lower GI damage remains common and often underdiagnosed, further contributing to the overall burden of NSAID-related toxicity (Bjarnason et al. 2018).

In this context, strategies aimed at reducing NSAID exposure while preserving analgesic efficacy are highly desirable. Our findings provide strong preclinical evidence supporting a dose-sparing approach based on the combination of meloxicam with PEAum, a small-particle, high bioavailable formulation of the endogenous prohomeostatic N-acylethanolamine PEA. Previous studies have shown that PEAum exhibits a superior absorption compared to unprocessed PEA and leads to significantly higher plasma levels in rats with carrageenan-induced inflammatory pain than healthy animals (Petrosino et al. 2018). These pharmacokinetic properties translate into faster and more pronounced antinociceptive effects compared with non-micronized formulations (Impellizzeri et al. 2014). Consistent with these observations, in the present study PEAum emerged as the most effective formulation when compared with micronized and native PEA, confirming the critical role of bioavailability in determining its pharmacodynamic profile.

A central implication of our findings is that PEAum can potentiate the analgesic efficacy of meloxicam. To our knowledge, this represents the first demonstration that the effect of an NSAID can be enhanced through co-administration of PEA. This finding aligns with, yet substantially expands, previous evidence showing that PEA enhances the efficacy of opioid analgesics, including tramadol, oxycodone (Micheli et al. 2022), and morphine, while simultaneously attenuating the development of tolerance. These effects are consistent with PEA’s role as a modulator of pain pathways rather than a simple analgesic agent (Di Cesare Mannelli et al. 2015, 2018). More recently, Nobili and colleagues comprehensively reviewed the capacity of PEA—particularly in its ultramicronized formulation—to act as an add-on therapy across multiple pharmacological classes, including paracetamol, tricyclic antidepressants, serotonin–norepinephrine reuptake inhibitors, and anti‑seizure agents such as gabapentin and pregabalin (Nobili et al. 2024). In addition, a steroid-sparing effect of PEAum has been suggested in feline allergic patients (Noli et al. 2019). Collectively, these findings position PEAum as a versatile, multimodal enhancer of analgesic efficacy, capable of improving outcomes across mechanistically diverse drug classes. Our results extend this paradigm to NSAIDs. Supporting this perspective, dietary supplementation with the co-micronized PEA analogue N-palmitoyl-D-glucosamine in dogs with osteoarthritis enabled a progressive reduction—and in some cases discontinuation—of meloxicam while maintaining effective pain control (Della Rocca et al. 2023).

A useful comparison can be made with the recent study by Xu and colleagues, who evaluated PEA-NSAID co‑prodrugs (in both crystal and nanocrystal formulations, 100–200 nm) in the CFA model of chronic pain (Xu et al. 2026). Two aspects of their findings are particularly relevant. First, no evidence of interaction, neither additive nor synergistic, was observed between PEA and naproxen; PEA alone, naproxen alone, and their combination produced comparable analgesic effects. This outcome may reflect the use of native PEA, whose limited bioavailability is known to constrain its pharmacological activity. Second, the authors reported no substantial improvement when the co-prodrug was administered as nanocrystals compared with crystals, suggesting that reducing particle size below the micrometric range does not necessarily translate into enhanced efficacy for this specific molecular construct. Overall, these findings indicate that the synergistic effect observed here with PEAum and meloxicam may not be generalizable to all PEA–NSAID combinations. Further studies systematically evaluating PEAum in combination with other NSAIDs are needed to determine whether synergy depends on NSAID class, COX‑selectivity, or other pharmacodynamic features.

Mechanistically, our findings suggest a functional interplay between COX‑dependent pathways and PEA‑mediated neuroimmune modulation. Meloxicam primarily exerts its effect through inhibition of cyclooxygenase (COX) enzymes and the consequent reduction of prostaglandin synthesis (Cheng et al. 2024), thereby limiting peripheral sensitization. In contrast, PEAum downmodulates neuroimmune responses, including mast cell activation and glial reactivity, and activates PPAR-α-dependent pathways (Palazzo et al. 2019; Petrosino and Schiano Moriello 2020; Di Stefano et al. 2025). The convergence of these mechanisms likely results in a broader suppression of the inflammatory cascade, ultimately leading to enhanced analgesic efficacy. Our pharmacodynamic data further support the involvement of PPAR-α in this interaction. Pretreatment with the selective PPAR-α antagonist GW6471 significantly reduced the antinociceptive effect of the PEAum- meloxicam combination in the LPS-inflammatory pain model. This indicates that the observed synergy is at least in part mediated by PPAR-α receptors.

From a translational perspective, the most clinically relevant implication of this study is that combining PEAum with meloxicam allows achieving full analgesic efficacy with a markedly reduced NSAID dose. This is particularly important in chronic conditions and in frail patients, where long-term NSAID use is associated with cumulative toxicity. The possibility of reducing meloxicam dosage by up to tenfold, while maintaining efficacy, underscores the strong clinical potential of this approach, especially in veterinary settings where meloxicam is extensively used.

In conclusion, our findings identify ultramicronized PEA as a potent adjuvant capable of enhancing meloxicam-induced analgesia through a synergistic interaction. By targeting complementary mechanisms involved in inflammatory pain, the PEAum-meloxicam combination represents a promising multimodal strategy for achieving safer and more effective pain management. Further studies are warranted to determine whether this enhancement of analgesic efficacy extends to other NSAIDs.

## Data Availability

The datasets obtained during the current study are available from the corresponding author on reasonable request due to concerns regarding data privacy.
